# Influence of day of surgery and prediction of LOS > 2 days after fast-track hip and knee replacement

**DOI:** 10.1080/17453674.2020.1844946

**Published:** 2020-11-12

**Authors:** Christoffer C Jørgensen, Kirill Gromov, Pelle B Petersen, Henrik Kehlet, on behalf of the Lundbeck Foundation Centre for Fast-track Hip and Knee Replacement Collaborative Group

**Affiliations:** a Section for Surgical Pathophysiology, Rigshospitalet, Copenhagen;; b Lundbeck Foundation Centre for Fast-track Hip and Knee Arthroplasty;; c Department of Orthopaedic Surgery, Clinical Orthopaedic Research Hvidovre (CORH), Copenhagen University Hospital Hvidovre, Denmark

## Abstract

Background and purpose — Enhanced recovery programs have reduced length of stay (LOS) after hip and knee arthroplasty (THA/TKA). Although risk factors disposing to prolonged LOS are well documented, there is limited information on the role of weekday of surgery. This study analyzed the role of weekday of surgery and other potential risk factors for LOS > 2 days.

Patients and methods — We included 10,576 unselected consecutive procedures between January 2016 and August 2017 within a multicenter fast-track THA/TKA collaboration with prospective collection of preoperative characteristics. We used multiple regression analysis of potential risk factors for LOS > 2 days followed by construction of a simple risk score from 0 to 15 points based on the calculated odds ratios.

Results — Mean LOS was 1.9 (SD 1.8) days, with 80% of patients having surgery from Monday to Wednesday. Of these, 17% (95% CI 16–18) had a LOS > 2 days vs. 19% (CI 17–21) in those operated on Thursday and Friday. Patients were scheduled evenly throughout the week regardless of risk of LOS > 2 days and despite the fact that 38% (CI 35–40) of patients with ≥ 6 points (16% of the total population) had a LOS > 2 days compared with 14% (CI 13–14) in those with < 6 points. In these “high-risk” patients, the fraction with LOS > 2 days increased when having surgery on Thursdays or Fridays (43% CI 38–49) compared with Monday to Wednesday (37% CI 34–39).

Interpretation — A detailed preoperative risk assessment may be helpful to plan the weekday of surgery in order to decrease LOS and weekend hospitalization.

Recent developments in perioperative care have also led to enhanced recovery (ERAS) in hip (THA) and knee replacement (TKA) with a decrease in postoperative length of stay (LOS) to between 0 and 2 days in many centers (Wainwright and Kehlet 2019). These advances have led to several studies showing the feasibility of outpatient THA and TKA in selected patients (Vehmeijer et al. 2018).

The positive effects of ERAS programs in THA and TKA remain indisputable, not only by reducing LOS, but also by lowering the risk of medical complications without an increase in readmissions (Wainwright and Kehlet 2019). However, challenges still exist to further improve outcome and where the strategy must be divided between optimizing preoperative comorbidities, perioperative care, and organizational issues, of which the latter has received less attention. Preoperative risk factors have been well assessed over several years in relation to short LOS and generally confirming increased age, obesity, diabetes, cardio-pulmonary diseases, and dependent functional status as risk factors for prolonged LOS (Jørgensen et al. 2016, Cram et al. 2018, Kim et al. 2018, Cizmic et al. 2019, Gkagkalis et al. 2019, Ziemba-Davis et al. 2019, Johnson et al. 2020). However, limited information is available from a fast-track setting on the role of weekday of surgery on LOS when adjusting for the above-mentioned risk factors. In this context, identification of patients unlikely to be discharged within 1–2 days and therefore to be scheduled for surgery at the start of the week may reduce the need for weekend hospitalization and transfer to other departments from otherwise well-functioning 5-day arthroplasty units or ambulatory arthroplasty centers.

The Lundbeck Foundation Centre for Fast-track Hip and Knee Arthroplasty (www.fthk.dk) was founded as a multi-institutional collaboration to improve care and outcome after THA and TKA and the most recent high-volume data have shown a median LOS of only 1 day in unselected patients (Petersen et al. 2019). The present study is a specific analysis of the role of preoperative risk factors and the weekday of surgery for a LOS > 2 days within the multicenter fast-track THA and TKA collaboration, in which unselected patients have been assessed in detail preoperatively and with complete registration of LOS in a socialized healthcare system where common practice in Denmark is discharge to home (Petersen et al. 2019). 

## Patients and methods

### Study design

This was a descriptive multicenter cohort study based on the Lundbeck Foundation Centre for Fast-track Hip and Knee Replacement Database (LCDB). The LCDB is a prospective database that records information on patient characteristics using patient-reported questionnaires. These are completed within 1 month prior to surgery with assistance from staff if necessary and a completeness of about 95% (Jørgensen et al. 2016). Currently 9 different departments report to the LCDB, all of which are dedicated arthroplasty units with > 300 annual procedures and similar fast-track protocols including preference for spinal anesthesia, multimodal opioid-sparing analgesia, in-hospital only thrombo-prophylaxis when LOS ≤ 5 days and early mobilization (≤ 6 hours postoperatively). High-dose methylprednisolone (125 mg) is standard in TKA and increasingly used in THA, while peripheral nerve blocks are only used at the discretion of the attending anesthesiologist. Readiness for discharge is evaluated using similar standardized functional discharge criteria including being able to get in and out of bed/chair, walking independently with an aid, and performing daily activities (Husted et al. 2010). There are no selection criteria for being included in the fast-track protocol as it is considered the standard of care in all participating departments (Petersen et al. 2019).

Data from the LCDB are cross-referenced with the Danish National Patient Registry, which records all hospitalizations in Denmark regardless of geographic location. The accuracy of the DNPR with regards to capturing admissions is > 99%, while the accuracy of individual diagnostic codes varies (Schmidt et al. 2015).

### Patients

**Figure 1.**
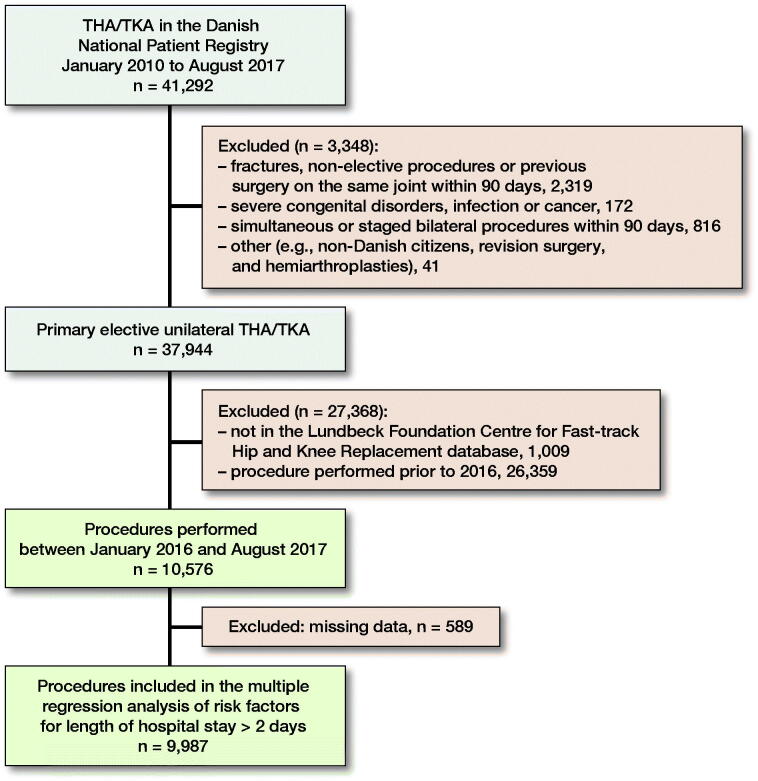
Study population. THA – total hip arthroplasty, TKA – total knee arthroplasty.

We included consecutive unselected unilateral THA and TKA from the LCDB between January 2016 and August 2017, excluding patients with age < 18 years, simultaneous bilateral procedures, procedures due to cancer or severe congenital disorder, and patients with major surgery on the lower extremities before or after 90 days from the elective procedure (Figure 1).

### Statistics

Comparison of categorical data was done using a chi-square test and reporting 95% confidence intervals (CI) for proportions. When testing for statistical significance a p-value of < 0.05 was used. Multiple logistic regression analysis was used for evaluating association between risk factors, weekday of surgery, and risk of LOS > 2 days. We included variables based on acyclic graphs to avoid mediation (Shrier and Platt 2008) and based on previous works (Jørgensen and Kehlet 2013b, Jørgensen et al. 2016). Finally, place of surgery was included as a random effect. We used complete case analysis as missing data was limited to about 5%. When evaluating the results of the risk score we included patients excluded from the regression model when possible. Model accuracy was reported as 83.3% using the Generalized Linear Mixed Model function in SPSS v. 25 (IBM Corp, Armonk, NY, USA).

When constructing a risk score for LOS > 2 days we assigned points for each significant variable in the regression model depending on the calculated ORs. Variables with an OR between 1 and 1.8 were assigned 1 point, ORs 1.9–2.9 were assigned 2 points and ORs > 2.9 were assigned 3 points. Thus, a score ranging from 0 to 15 was constructed (see Results section). We evaluated the fit of the risk score using an ROC curve and based a cut-off for high-risk patients based on the calculated sensitivity and specificity, as well as consideration of clinical relevance.

### Ethics, registration, funding, and potential conflicts of interest

As this is a non-interventional study, ethical approval was waived, but permission to collect and store data was obtained from the Danish Patient Safety authority (3-3013-56/2/EMJO) and the Danish Data Protection Agency (2012-58-0004). The LCDB is registered on clinicaltrials.gov (NCT01515670) as an ongoing registry study on outcomes after fast-track THA and TKA. The LCDB has been funded by a grant from the Lundbeck Foundation (R25-A2702). The Lundbeck Foundation is independent of the Lundbeck Pharmaceutical company and had no influence on any part of the study design or writing of the manuscript.

CJ and PB declare no conflicts of interest. KG and HK are members of the board on “Rapid Recovery” by Zimmer Biomet.

## Results

Of 10,576 procedures, 9,987 (94%) could be included in the multiple regression analysis (Figure 1 and Table 1). Median LOS was 2 (IQR 1) days and mean 1.9 day (SD 1.8).

**Table 1.** Patient characteristics Values are count (%) unless otherwise specified

Factor	n (%)
Age median (IQR)	70 (14)
< 50	514 (4.9)
50–60	1,671 (16)
61–65	1,384 (13)
66–70	2,055 (19)
71–75	2,218 (21)
76–80	1,639 (16)
81–85	806 (7.6)
> 85	289 (2.7)
BMI median (IQR)	27.4 (6.5)
< 18.5	94 (0.9)
18.5–24.9	4,110 (39)
25–29.9	2,968 (28)
30–34.9	2,237 (21)
35–39.9	788 (7.5)
> 39.9	279 (2.7)
Missing	100 (0.9)
Female sex	6,213 (59)
Walking aid	2,391 (23)
Missing	195 (1.8)
Living alone	3,632 (35)
In institution	75 (0.7)
Missing	80 (0.8)
Smoking	1,347 (13)
Missing	89 (0.8)
Alcohol > 24 g/day	790 (7.5)
Missing	91 (0.9)
Total knee arthroplasty	4,448 (42)
Psychiatric disease	1,520 (14)
Missing	0 (0.0)
Cardiac disease	1,439 (14)
Missing	106 (1.0)
Pulmonary disease	945 (9.0)
Missing	64 (0.6)
Hypertension	5,911 (56)
Missing	0 (0.0)
Diabetes mellitus	
Non-insulin dependent	912 (8.8)
Insulin dependent	199 (1.9)
Missing	61 (0.6)
Anticoagulants	821 (7.8)
Missing	0 (0.0)
Preoperative anemia **^a^**	2,522 (24)
Missing	155 (1.5)

**^a^** Hemoglobin < 13 g/dL.

**Figure 2.**
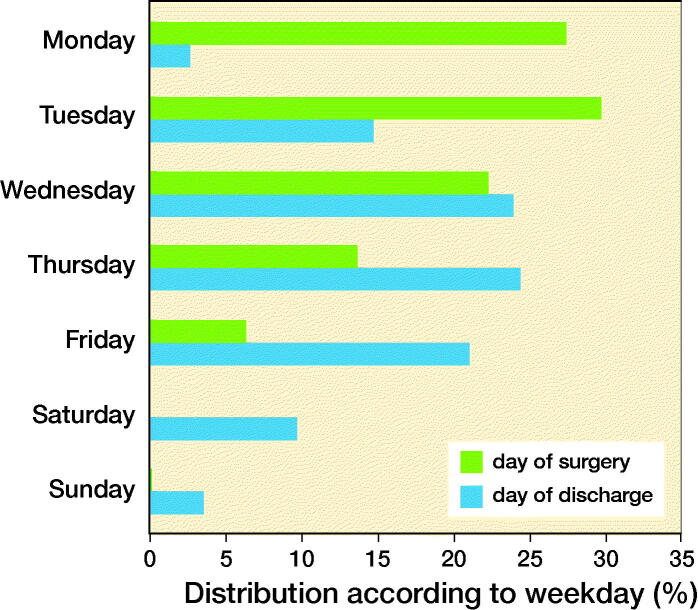
Distribution of procedures and day of discharge according to weekday. There were 11 (0.1%) procedures on Saturday and Sunday, all in patients with < 6 points.

The distribution of weekday of surgery was left skewed with > 50% of all procedures being performed on Mondays (28%) and Tuesdays (30%). Only 14% and 6% of procedures were performed on Thursdays and Fridays, respectively. Finally, there were 11 (0.1%) procedures performed during the weekend, all in patients later found to be at low risk (score < 6, see below) of having LOS > 2 days. These patients were subsequently excluded from the weekday analysis as they would probably have been selected to be performed on special personal indication (Figure 2). The number of THAs or TKAs performed on Monday to Wednesday (79% vs. 80%) or Thursday to Friday (21% vs. 20% in THA and TKA, respectively) was similar. The weekday of discharge was peaking on Thursdays, but with a considerable number of patients being discharged during the weekend (13%) (Figure 2).

When analyzing the influence of weekday of surgery on risk of having a LOS > 2 days it was possible to include 9,987 (94%) procedures (Figure 1). Only surgery on a Friday was associated with a significantly higher risk of LOS > 2 days after adjusting for patient characteristics (Table 2). The proportion of patients who had their operation on Mondays, Tuesdays, or Wednesdays was 80%, of whom 17% (CI 17–18) had a LOS > 2 days compared with 19% (CI 17–21) with LOS > 2 days in those having surgery on Thursday to Friday (OR 1.1).

**Table 2.** Multiple-regression analysis of risk factors (n = 9,987) for LOS > 2 days and attributed risk-score points

Variable	OR (95%CI)	p-value	points
Weekday of surgery			
Monday	1 (ref.)	–	
Tuesday	1.0 (0.9–1.2)	1.0	
Wednesday	0.9 (0.7–1.0)	0.08	
Thursday	0.9 (0.8–1.1)	0.4	
Friday	1.5 (1.2–2.0)	< 0.01	
Saturday	2.9 (0.3–30.5)	0.4	
Sunday	1.4 (0.2–13.6)	0.8	
Age group			
< 50	1.3 (0.9–1.8)	0.08	
50–60	1.2 (0.9–1.4)	0.1	
61–65	1.0 (0.8–1.3)	0.9	
66–70	1 (ref.)	–	
71–75	1.2 (1.0–1.4)	0.1	
76–80	1.4 (1.2–1.7)	< 0.01	1
81–85	2.0 (1.6–2.5)	< 0.01	2
> 85	3.4 (2.5–4.6)	< 0.01	3
BMI			
< 18.5	1.6 (1.0–2.6)	0.07	0
18.5–24.9	1 (ref.)	–	
25–29.9	1.0 (0.8–1.1)	0.7	0
30–34.9	1.0 (0.9–1.2)	1.0	0
35–39.9	1.2 (0.9–1.5)	0.2	0
> 39.9	1.3 (0.9–1.8)	0.1	
Female sex	1.3 (1.2–1.5)	< 0.01	1
Walking aid	1.9 (1.7–2.3)	< 0.01	2
Living alone	1.6 (1.4–1.8)	< 0.01	1
Smoking	1.1 (0.9–1.3)	0.5	0
Alcohol > 24 g/day	0.8 (0.6–1.0)	0.06	0
Total knee arthroplasty	1.9 (1.7–2.1)	< 0.01	1
Psychiatric disease	1.6 (1.4–1.9)	< 0.01	1
Cardiac disease	1.2 (1.0–1.4)	0.04	1
Pulmonary disease	1.3 (1.1–1.6)	< 0.01	1
Hypertension	1.0 (0.9–1.1)	0.8	0
Diabetes mellitus			
Non-insulin dependent	1.3 (1.1–1.6)	0.02	1
Insulin dependent	2.0 (1.4–2.9)	< 0.01	2
Anticoagulants	1.2 (1.0–1.5)	0.09	0
Preoperative anemia **^a^**	1.5 (1.3–1.7)	< 0.01	1

OR: odds ratio. CI: confidence interval.

**^a^** Hemoglobin < 13 g/dL.

Multiple logistic regression on preoperative risk factors associated with a LOS > 2 days was possible in 9,987 patients, and found association with age (76–80: OR 1.4, 81–85: OR 2.0, and > 85: OR 3.4), female sex (OR 1.3), use of walking aids (OR 1.9), living alone (OR 1.6), TKA (OR 1.9), psychiatric disorder (OR 1.6), cardiac (OR 1.2) or pulmonary disease (OR 1.3), both non-insulin (OR 1.3) and insulin-dependent diabetes (OR 2.0), and preoperative anemia (OR 1.5) (Table 2).

**Figure 3.**
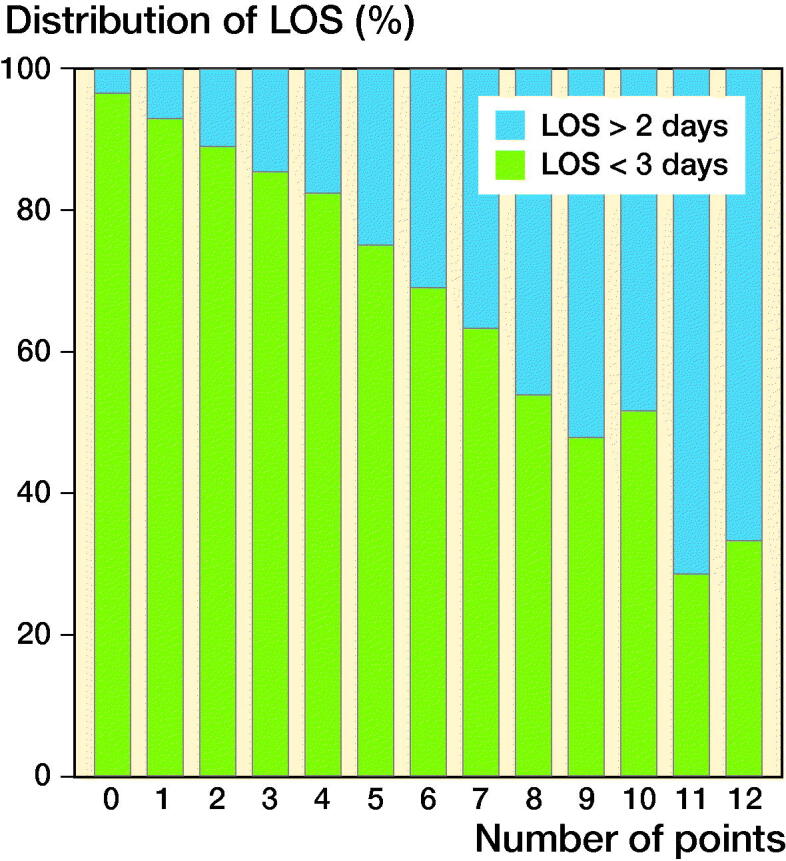
Distribution of patients with length of stay (LOS) > 2 days and < 3 days according to number of points based on odds ratios of relevant risk factors for LOS > 2 days.

**Figure 4.**
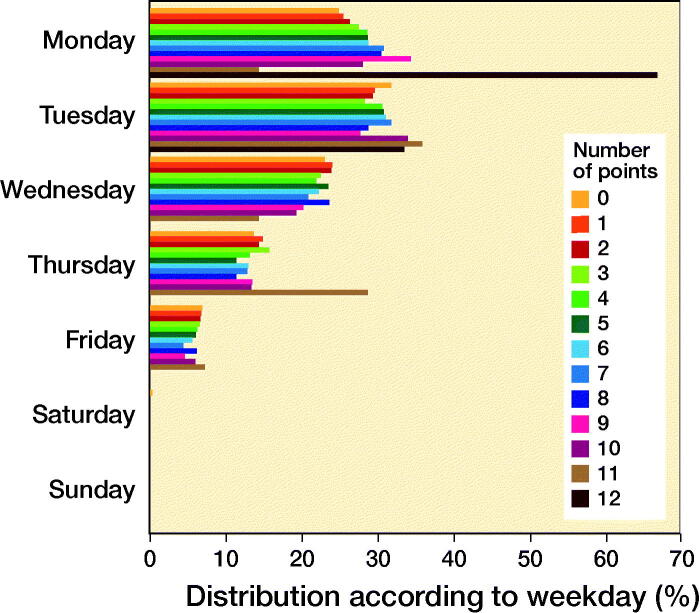
Distribution of procedures on each weekday according to number of points for LOS > 2 days.

After assigning values to the significant risk factors for LOS > 2 days according to their respective ORs, a score ranging from 0 to 15 was constructed with increasing points indicating increasing risk of LOS > 2. However, only 3 patients scored 12 points and no patient scored more than 12 points (Table 3). An ROC curve was constructed for this model showing an area under the curve of 0.70 (CI 0.69–0.72). The fraction of patients with LOS > 2 days increased with increasing scores (Figure 3), and there was an even distribution of patients across weekdays regardless of score (Figure 4). When deciding on an adequate cut-off for at-risk patients, a threshold of ≥ 7 points would yield a sensitivity of 23% and a specificity of 93%. However, such patients attributed only 11% of the total population, severely reducing clinical relevance. Correspondingly, a threshold of ≥ 6 points would increase sensitivity (36%), decrease specificity (87%), and include 17% of the total population. Finally, sensitivity would increase to 51% and specificity decline to 78% but include 28% of all patients if using a threshold of ≥ 5 points. Consequently, a threshold of ≥ 6 points was chosen as this would yield acceptable sensitivity and specificity while including a clinically relevant and manageable number of patients. Of the “high-risk patients” with ≥ 6 points, 38% (n: 635) had a LOS > 2 and median LOS was 4 days (IQR 2). 18% had surgery on either Thursdays or Fridays of whom 43% (CI 38–49) stayed > 2 days compared with 37% (CI 34–39) who had surgery on Monday through Wednesday (OR 1.3, CI 1.0–1.7; p = 0.04). In contrast, 14% (CI 13–14) of patients with a risk score of < 6 had a LOS > 2 days and a median LOS of 3 days (IQR 1).

**Table 3.** Distribution of points depending on number of risk factors for LOS > 2 days

Number of points	n (%)
0	898 (8.9)
1	1,278 (13)
2	1,759 (17)
3	1,902 (19)
4	1,497 (15)
5	1,107 (11)
6	727 (7.2)
7	449 (4.4)
8	293 (2.9)
9	134 (1.3)
10	68 (0.7)
11	14 (0.1)
12	3 (0.0)
13	0 (0)
14	0 (0)
15	0 (0)

N = 10,129 due to 447 cases with missing data for variables associated with LOS > 2 days (see Table 1).

## Discussion

Although ERAS programs have been documented to decrease LOS after THA and TKA in many centers to median 0 to 2 days, limited information is available on the influence of specific risk factors and weekday of surgery on LOS. Thus, to our knowledge, only 1 previous retrospective single-center study on TKA has investigated the influence of weekday of surgery and found increased risk of LOS > 3 days when having surgery on Thursdays (Mathijssen et al. 2016). Consequently, we used prospective data collected from the well-established Lundbeck Foundation Centre for Fast-track Hip and Knee Replacement database to study the association between preoperative risk factors, weekday of surgery and LOS > 2 days. The results of our analysis, which found anemia, use of walking aids, age > 75 years, TKA, female sex, living alone, psychiatric, pharmacologically treated cardiac and pulmonary disease, and type of diabetes to be important risk factors, are unsurprising as they have previously been demonstrated to influence LOS > 4 days in a fast-track setup (Jørgensen and Kehlet 2013b, Jans et al. 2014, Jørgensen et al. 2016, Cram et al. 2018, Johnson et al. 2020). One of the reasons that TKA was a risk factor for LOS > 2 days may be related to more extensive pain problems compared with THA, consequently prolonging time to achieving functional discharge criteria. However, TKA has also been found to be an independent predictor of potentially preventable “surgical” complications, which mostly occur after discharge, mainly prosthetic infections and manipulation under anesthesia (Jørgensen et al. 2016). That female sex was a risk factor has been found in some (Winemaker et al. 2015) but not in all studies (den Hartog et al. 2015, Jørgensen et al. 2016). A recent review specifically on TKA also found female sex to be a risk factor for increased LOS (Shah et al. 2019). However, although several of the included studies utilized “fast-track” programs, reported LOS was longer than in our study (3 days or more). In contrast, conventional risk factors such as BMI, smoking, and alcohol use have often been reported to influence complications and LOS (Belmont et al. 2014, Best et al. 2015, Winemaker et al. 2015, Jeschke et al. 2018, Sahota et al. 2018), but may be of reduced importance within a fast-track setup (Jørgensen et al. 2013a, den Hartog et al. 2015, Husted et al. 2016). Importantly, as pointed out by Shah et al., the influence of a single risk factor is often clinically negligible, but the combination of several risk factors may increase patients’ disposition for extended LOS (Shah et al. 2019). This is further illustrated by the ideal threshold for defining “high-risk” patients being about 6 points on our risk score. Regarding the point score for identification of patients at high risk of having a LOS > 2 days the primary objective of our study was not to construct the ideal final risk-prediction model for LOS > 2 days. Rather, we wanted to investigate whether it would be possible to provide a simple algorithm for identification of patients who may benefit from having surgery early in the week. Consequently, we chose a pragmatic approach that included only significant risk factors and assigned increasing points according to increasing odds ratios. Thus, the presented THA/TKA risk score may likely benefit from further optimization to increase sensitivity and specificity, but bearing in mind that it should be effective, simple, and fast to use in clinical practice. In this context the clinical influence of advanced risk calculators such as the NSQUIP surgical-risk calculators on surgical planning and postoperative outcomes remains uncertain, potentially due to difficulties with implementation in clinical practice (Moonesinghe et al. 2013).

The finding that patients with ≥ 6 points had an even higher risk of LOS > 2 days when having surgery on Thursday or Friday is not unexpected when considering the reduction in availability of staff resources and other providers (e.g., primary care providers etc.) during the weekend, although we have no data to confirm this suspicion. One could speculate that other logistic issues would influence whether patients were discharged earlier, i.e., premature discharge in the middle of the week or longer admission at the weekend due to beds being available. However, all departments use functional discharge criteria, and transfer to rehabilitation homes is extremely rare, occurring in < 7% of patients aged ≥ 85 years (Pitter et al. 2016). Thus, it seems unlikely that patients would be discharged prematurely in the middle of the week in order to free up beds. Furthermore, the proportion of patients being discharged on Saturdays largely reflected the number of surgeries on Thursdays, arguing against unnecessary prolongation of admission over the weekend.

However, our results may be of clinical relevance, as the proposed risk score may be useful in planning weekday of surgery, potentially reducing the risk of LOS extending into the weekend. This would be of special value in ambulatory surgical centers or 5-day surgical units. Interestingly, although the Lundbeck Foundation Centre for Fast-track THA and TKA has been documented to be successful in reducing LOS (Petersen et al. 2019), apparently the present data analysis of risk factors vs. choice of weekday of surgery has not been included in the daily logistical preparation within the centers where about 20% of operations were performed on Thursdays and Fridays but with an even distribution of “high-” and “low-”risk patients throughout the week.

With regard to the definition of LOS, our study calculates LOS as postoperative nights in hospital and where the most recent data have shown a mean LOS of 1.9 days in unselected patients. (Petersen et al. 2019). In contrast, there are several studies where outpatient procedures are defined as less than 24 hours or less than 2 midnights (Vehmeijer et al. 2018, Johnson et al. 2020) or with use of nursing care facilities or rehabilitation homes (Cram et al. 2018, Ross et al. 2020), which may give a false impression of reduced LOS. In this context, a limitation of our study is the lack of detailed information on discharge destination although non-home discharge after fast-track THA and TKA is limited in Denmark and even in patients > 85 years been demonstrated to occur in only about 7% of patients (Pitter et al. 2016). Furthermore, we do not have detailed data on why patients were admitted for > 2 days or whether they were transferred to other departments. Such data would be of interest in order to define the exact number of patients remaining in the arthroplasty departments during the weekend, and how to prevent it. The strengths of our study are detailed prospective registration of risk factors, complete data on index LOS, and a large cohort of non-selected THA and TKA patients from a well-established enhanced recovery multicenter collaboration developed across the last decade.

In summary, the planning of day of surgery in relation to relevant preoperative risk factors should receive more attention and may lead to an overall decrease in LOS and consequently risk of hospitalization into the weekend in high-risk patients. 
